# miR-15a/miR-16-1 expression inversely correlates with cyclin D1 levels in *Men1* pituitary NETs

**DOI:** 10.1530/JOE-18-0278

**Published:** 2018-09-28

**Authors:** K E Lines, P J Newey, C J Yates, M Stevenson, R Dyar, G V Walls, M R Bowl, R V Thakker

**Affiliations:** 1 Academic Endocrine Unit, Radcliffe Department of Medicine, University of Oxford, Oxford Centre for Diabetes, Endocrinology and Metabolism (OCDEM), Churchill Hospital, Headington, Oxford, UK; 2 Division of Molecular & Clinical Medicine, University of Dundee, Ninewells Hospital & Medical School, Dundee, UK

**Keywords:** microRNA, neuroendocrine tumour, menin, multiple endocrine neoplasia type 1

## Abstract

Multiple endocrine neoplasia type 1 (MEN1) is an autosomal dominant disorder characterised by the combined occurrence of parathyroid, pituitary and pancreatic islet tumours, and is due to mutations of the *MEN1* gene, which encodes the tumour suppressor protein menin. Menin has multiple roles in genome stability, transcription, cell division and proliferation, but its mechanistic roles in tumourigenesis remain to be fully elucidated. miRNAs are non-coding single-stranded RNAs that post-transcriptionally regulate gene expression and have been associated with tumour development, although the contribution of miRNAs to MEN1-associated tumourigenesis and their relationship with menin expression are not fully understood. Alterations in miRNA expression, including downregulation of three putative ‘tumour suppressor’ miRNAs, miR-15a, miR-16-1 and let-7a, have been reported in several tumour types including non-MEN1 pituitary adenomas. We have therefore investigated the expression of miR-15a, miR-16-1 and let-7a in pituitary tumours that developed after 12 months of age in female mice with heterozygous knockout of the *Men1* gene (*Men1^+/^^−^* mice). The miRNAs miR-15a, miR-16-1 and let-7a were significantly downregulated in pituitary tumours (by 2.3-fold, *P* < 0.05; 2.1-fold *P* < 0.01 and 1.6-fold *P* < 0.05, respectively) of *Men1^+/^^−^* mice, compared to normal WT pituitaries. miR-15a and miR-16-1 expression inversely correlated with expression of cyclin D1, a known pro-tumourigenic target of these miRNAs, and knockdown of menin in a human cancer cell line (HeLa), and AtT20 mouse pituitary cell line resulted in significantly decreased expression of miR-15a (*P* < 0.05), indicating that the decrease in miR-15a may be a direct result of lost menin expression.

## Introduction

Multiple endocrine neoplasia type 1 (MEN1) is an autosomal dominant disorder characterised by the combined occurrence of parathyroid, pituitary and pancreatic islet tumours (Pieterman *et al.* 2009, Goudet *et al.* 2010, Thakker *et al.* 2012, Frost *et al.* 2018). MEN1 is due to mutations of the *MEN1* gene, which encodes the tumour suppressor protein menin (Chandrasekharappa *et al.* 1997, Concolino *et al.* 2016, Lemos & Thakker 2008, Lemmens *et al.* 1997). Loss of menin expression is observed in the majority of MEN1-associated tumours, in keeping with Knudson’s two-hit model of inherited tumourigenesis (Chandrasekharappa *et al.* 1997, Concolino *et al.* 2016, Lemos & Thakker 2008, Lemmens *et al.* 1997). Menin is involved in a diverse range of cellular processes including: transcriptional regulation, genome stability, cell division and proliferation (Thakker *et al.* 2012, Frost *et al.* 2018). However, the mechanisms by which menin loss results in tumourigenesis are not fully understood. One mechanism that is likely to be implicated involves miRNAs, which have been reported to have roles in the development of a large number of other tumour types (Filipowicz *et al.* 2008, Stefani & Slack 2008). Moreover, menin has been reported to regulate the expression of miRNAs (Luzi *et al.* 2012*a*,*b*, Wang *et al.* 2013, Gurung *et al.* 2014, Li *et al.* 2014, Ouyang *et al.* 2015, Ehrlich *et al.* 2017, Hou *et al.* 2017), which are short, non-coding, single-stranded RNAs that post-transcriptionally regulate gene expression, predominantly by imperfect base pairing to the 3′ untranslated region (UTR) of target mRNA sequences (Filipowicz *et al.* 2008, Stefani & Slack 2008). The importance of miRNAs has been illustrated by their ability to influence a wide spectrum of cellular processes including proliferation, apoptosis and differentiation, in a tissue-specific manner and many miRNAs have been implicated in tumour development through the ability to influence the expression of a diverse set of target genes, including tumour suppressors and oncogenes (Peng & Croce 2016).

The role of miRNAs in pituitary tumourigenesis has been investigated by microarray-based profiling studies, which have revealed changes in their expression (Wierinckx *et al.* 2017). For example, sporadic human pituitary tumours have been reported to have altered expression of multiple miRNAs, when compared to normal pituitary tissue (Bottoni *et al.* 2005, 2007, Amaral *et al.* 2009, Qian *et al.* 2009, D’Angelo *et al.* 2012, Palmieri *et al.* 2012). The functional significance of such changes in miRNA expression in endocrine tumourigenesis, especially in relation to MEN1-associated pituitary tumours remains unknown. It has however been demonstrated that the putative tumour suppressor miRNAs miR-15a, miR-16-1 and let-7 are downregulated in non-functioning adenomas, prolactinomas, somatotrophinomas and corticotrophinomas and that loss of miR-15a and miR-16-1 correlates with increased tumour diameter, while loss of let-7 expression is correlated with increased tumour grade (Bottoni *et al.* 2005, 2007, Amaral *et al.* 2009, Qian *et al.* 2009, D’Angelo *et al.* 2012, Palmieri *et al.* 2012). The specific genetic targets of these miRNAs in pituitary neuroendocrine tumours have not been elucidated, but studies in chronic lymphocytic leukaemia (CLL) have shown that loss of miR-15a and miR-16-1, which co-occur as a cluster, allows overexpression of BCL2, while investigations in prostate and non-small-cell lung cancer have reported that downregulation of miR-15a and miR-16-1 results in increased cyclin D1 (*CCND1*) expression, with each contributing to tumour formation (Cimmino *et al.* 2005, Bonci *et al.* 2008, Calin *et al.* 2008, Bandi *et al.* 2009, Croce 2009, Salerno *et al.* 2009). Similarly, in non-endocrine tumours members of the let-7a family have been shown to target the oncogenes *KRAS*, *MYC* and *HMGA2* (Johnson *et al.* 2005, Mayr *et al.* 2007, Kumar *et al.* 2008) in a tumour-specific manner. In addition, menin has been reported to directly regulate the expression of miRNA genes via its role as a transcriptional regulator or through miRNA processing (Luzi *et al.* 2012*a*,*b*, Wang *et al.* 2013, Gurung *et al.* 2014, Li *et al.* 2014, Ouyang *et al.* 2015, Ehrlich *et al.* 2017, Hou *et al.* 2017), although the role of miRNAs in MEN1-associated tumours remains to be established. Therefore to determine if the reported downregulation of miR-15a, miR-16-1 and let-7a in pituitary tumours is a result of loss of menin expression, we investigated the role of menin in the regulation of miR-15a, miR-16-1 and let-7a, using a previously reported murine model of MEN1 (Harding *et al.* 2009), the mouse pituitary cell line AtT20 and a human cell-based assay that utilised the human cervical adenocarcinoma (HeLa) cell line.

## Materials and methods

### Generation of *Men1^+/−^* mice

Animal studies were approved by the University of Oxford Ethical Review Committee and were licensed under the Animal (Scientific Procedures) Act 1986, issued by the United Kingdom Government Home Office Department (PPL30/2914). A conventional *Men1*-knockout model generated by targeted deletion of exons 1 and 2 of the *Men1* allele was used (Harding *et al.* 2009, Lemos *et al.* 2009). The *Men1^+/^^−^* mice have been reported to develop parathyroid, pancreatic islet, anterior pituitary, adrenal cortical and gonadal tumours (Harding *et al.* 2009). This model was selected for investigation as greater than 40% of female mice (over the age of 12 months) develop discrete anterior pituitary tumours readily identifiable at autopsy (Harding *et al.* 2009). Genotypes of mice were determined by PCR analysis using DNA extracted from ear biopsies and *Men1* gene-specific primers, as previously reported (Lemos *et al.* 2009). Primers Men1F (5′-TAGATGTAGCTGGATGGTGATGG-3′) and Men1R (5′-ATGAAGCTGAGGAGATGATGTAG-3′) yielded a 582 base-pair WT fragment and primers Men1F and NeoR (5′-GCTGACCGCTTCCTCGTG-3′) yielded a 809 base-pair mutant fragment (Supplementary Fig. 1A, see section on supplementary data given at the end of this article). The mice were fed a standard diet (Rat and Mouse No. 1 expanded diet, Special Diet Services Ltd.), provided with water *ad libitum*, and weighed regularly. Pituitaries were isolated from five tumour-bearing female *Men1^+/^^−^* and five female WT (*Men1^+/+^*) mice, aged over 12 months and both maintained on a C57BL/6 background, for miRNA analysis. The study was limited to female mice because <5% of *Men1^+/^^−^* males develop pituitary tumours (Harding *et al.* 2009). The pituitary tumours from *Men1^+/^^−^* mice in this cohort, as previously reported (Harding *et al.* 2009), had loss of menin expression, compared to WT pituitaries isolated from *Men1^+/+^* mice (Supplementary Fig. 1B). Both pituitary tumours and WT pituitaries expressed prolactin (Supplementary Fig. 1B).

### Cell lines

Human *MEN1*-associated pituitary tumour cell lines or normal human pituitary cell lines are not available, and we therefore used the HeLa cell line to investigate the relationship between miR-15a, miR-16-1, cyclin D1 and *Men1*, as these cells express both miR-15a and miR-16-1, as well as menin and cyclin D1 (Supplementary Fig. 2), and a previous study has mapped the genomic binding sites of menin in these cells (Scacheri *et al.* 2006). HeLa cells (#CCL-2) and AtT20 cells (#CCL-89) were purchased from ATCC and used up to passage eight from the original stock. Both cell lines were maintained in Dulbecco’s Modified Eagle medium (DMEM), supplemented with 100 U/mL penicillin, 100 μg/mL streptomycin, 2 mM l-glutamine and 10% heat-inactivated foetal calf serum at 37°C, 5% CO_2_ and 95% humidity.

### Antagomir transfections

MicroRNA inhibitors (‘antagomirs’) to miR-15a and miR-16-1, as well as the control antagomir miR-1, were custom designed and purchased from Thermo Scientific (Table 1). Antagomirs are engineered oligonucleotides that competitively bind to a target miRNA and inhibit their activity. HeLa cells were seeded in six-well plates and transfected with 100 nM antagomirs diluted in serum free DMEM, using Dharmafect 1 transfection reagent (Thermo Scientific). Forty-eight hours post transfection cells were harvested for further analysis.

**Table 1:** Antagomir sequence.

Antagomir	Sequence
miR-15a	5′-mC(*)mA(*)mCmAmAmAmCmCmAmUmUmAmUmGmUmGmCmUmG(*)mC(*)mU(*)mA(*)-Chol-3′
miR-16-1	5′-mC(*)mG(*)mCmCmAmAmUmAmUmUmUmAmCmGmUmGmCmUmG(*)mC(*)mU(*)mA(*)-Chol-3′
miR-1 (Control)	5′-mA(*)mU(*)mAmCmAmUmAmCmUmUmCmUmUmUmAmCmAmUmU(*)mC(*)mC(*)mA(*)-Chol-3′

Specific mature mRNA binding oligonucleotide sequences, antagomirs, were designed to inhibit miR-15a and miR-16-1 activity by preventing the interaction of the miRNA with its mRNA seed sequence. A control miRNA, miR-1, was also designed. All antagomirs contain internal modifications to protect them from RNase-mediated degradation.

(*): Indicates a phosphorothioate linkage; ‘m’ indicates 2′-O-methyl modified nucleotides; ‘Chol’ represents a cholesterol group.

### siRNA transfection

Cells were seeded in six-well plates and transfected with 25 nM of either control, non-targeting (NT) siRNA or a species specific ON-TARGETplus SMARTpool of siRNAs against *MEN1*, using Dharmafect 1 transfection reagent (all Thermo Scientific) prepared in serum-free DMEM. After addition of siRNA, cells were incubated for 48 h and miRNA or protein harvested for further analysis.

### Quantitative reverse-transcriptase PCR (qRT-PCR)

Total RNA, including the miRNA fraction, was extracted from both mouse tissues and cell lines using the mirVana miRNA Isolation Kit (Ambion) according to the manufacturer’s instructions, and as previously described (Ma *et al.* 2007). RNA quality was determined by a NanoDrop ND-1000 Spectrophotometer (NanoDrop Technologies) and agarose gel electrophoresis. Up to 1 µg of total RNA was subsequently converted to cDNA using the miScript RT II kit (Qiagen), with HiFlex buffer (Qiagen) and qRT-PCR reactions were performed using the miScript SYBR green kit, according to the manufacturer’s instructions, on a Rotor-Gene Q Cycler (Qiagen). Human and mouse-specific miScript primer assays (Qiagen) were purchased for all miRNAs, and human and mouse specific QuantiTect primer assays (Qiagen) for all mRNAs. For all miRNA experiments data was normalised to the small nucleolar reference RNAs RNU6B and SNORD95, and for all mRNA experiments, data were normalised to the control mRNA GAPDH. The relative expression of target cDNA in all qRT-PCR studies was determined using the Pfaffl method, as previously described (Lines *et al.* 2017).

### Western blot

Cell lines and mouse pituitary tissues were lysed in NP40 lysis buffer: 250 mM NaCl, Tris 50 mM (pH 8.0), 5 mM EDTA, 0.5% NP-40 (v/v) and 2× Protease inhibitor tablets (Roche). Pituitary tissue samples were removed from −80°C storage immediately prior to use and homogenised in an appropriate volume of ice-cold NP40 lysis buffer. Cell lines were washed in PBS and each well lysed in 500 μL ice-cold NP40 buffer. Samples were incubated on ice for 10 min, centrifuged for 10 min at 96,000 ***g*** and the supernatant removed for analysis. Lysates were prepared in 5× Laemmli loading dye: 45 mmol/L Tris (pH 6.8), 10% glycerol, 1% SDS, 50 mmol/L DTT, 0.01% bromophenol blue, boiled at 95°C for 5 min and resolved using SDS-PAGE gel electrophoresis. Samples were transferred onto PVDF membrane (PerkinElmer), blocked in 5% Marvel (powdered-milk) in TBS-T and incubated with 1:100 rabbit anti-cyclin D1 antibody (AbCam), 1:1000 rabbit anti-menin antibody (Bethyl Laboratories) or 1:1000 rabbit anti-α-tubulin (AbCam) at 4°C overnight. Membranes were subsequently incubated with appropriate HRP-conjugated secondary antibodies and visualised using ECL Western blotting substrate (BioRad) on a BioRad Chemidoc XRS+ system and densitometric analysis performed using Image J, as previously described (Lines *et al.* 2017).

### Histology and immunohistochemistry

Pituitary tissues were dissected from mice, fixed with 4% paraformaldehyde, embedded in paraffin, and 4 μM sections dewaxed and hydrated for staining, as described (Lines *et al.* 2017). Sections were stained with haematoxylin and eosin, as previously described (Walls *et al.* 2016) or used for immunohistochemical staining, in which heat-mediated antigen retrieval was performed in citrate buffer and blocking in 10% donkey serum, before primary antibody incubation. Primary antibodies included rabbit anti-menin (ab2605 (AbCam)), and rabbit anti-prolactin (National Hormone and Pituitary Programme (NHPP)). The secondary antibody was horseradish peroxidase-conjugated goat anti-rabbit (Dako), visualised with a peroxidase/3,3′-diaminobenzidine Envision detection system (Dako). Nuclear counterstaining was performed with haematoxylin QS (Vector Laboratories). Sections were viewed by light or fluorescent microscopy using an Eclipse E400 microscope (Nikon), utilising a DXM1200Cdigital camera and NIS-Elements BR 2.30 software (both Nikon), as described (Lines *et al.* 2017).

### Statistical analysis

Data were analysed using Students *t*-test or one-way ANOVA using a Bonferroni correction for multiple comparisons, as previously described (Walls *et al.* 2012, Gorvin *et al.* 2013, Lines *et al.* 2017).

## Results

### miR-15a, miR-16-1 and let-7a expression are reduced in *Men1**^+/−^*** **mouse pituitary tumours**

**Figure 1:**
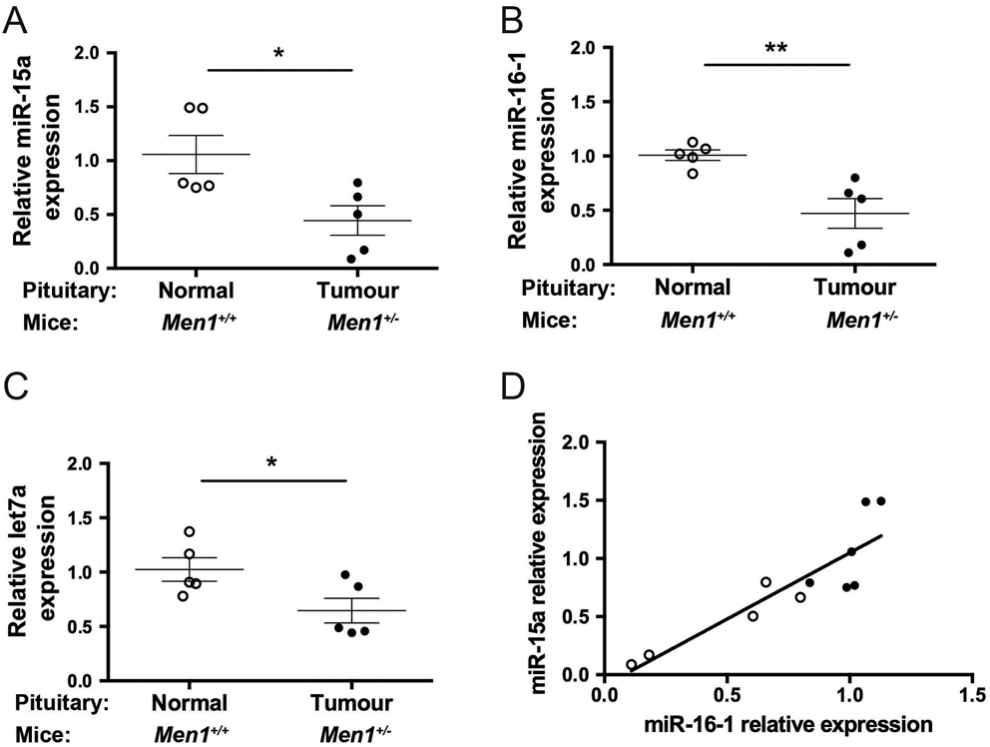
miR-15a, miR-16-1 and let-7a expression in pituitary tumours developing in *Men1^+/^^−^* mice. The expression of miR-15a, miR-16-1 and let-7a was compared in five pituitary tumours (closed circles) from *Men1^+/^^−^* female mice to five normal pituitaries (open circles) from age- and sex-matched WT *Men1^+/+^* control mice, using qRT-PCR. The mean and standard error of the mean are shown and samples were normalised to WT, which was set at 1. Expression of miR-15a (A), miR-16-1 (B) and let-7a (C) were each significantly decreased in pituitary tumour samples from *Men1^+/^^−^* mice compared to normal pituitary samples from *Men1^+/+^* mice; *P < 0.05, **P < 0.005. A significant positive correlation was also observed between the relative expression of miR-15a and miR-16-1 in the ten *Men1*-associated pituitary samples (tumour (closed circles) *n* = 5, and normal (open circles) *n* = 5) consistent with transcription from the same polycistronic cluster (D, *R* ^2^ = 0.77, *P* < 0.001).

Quantitative RT-PCR analysis of pituitary tumours isolated from *Men1^+/^^−^* mice or normal pituitary tissue isolated from WT mice, revealed the pituitary tumours, when compared to normal pituitaries, to have a significant decrease in the expression of miR-15a (2.3-fold, *P* < 0.05), miR-16-1 (2.1-fold *P* < 0.01) and let-7a (1.6-fold *P* < 0.05) (Fig. 1A, B and C). This observed downregulation of miR-15a, miR16-1 and let-7a in the *Men1^+/^^−^* pituitary tumours was consistent with their reported reduced expressions in human pituitary tumours (Bottoni *et al.* 2005, 2007, Amaral *et al.* 2009, Qian *et al.* 2009, D’Angelo *et al.* 2012, Palmieri *et al.* 2012). miR-15a and miR-16-1 are transcribed from the same polycistronic cluster, and an analysis of these miRNAs in the ten individual samples, demonstrated a significant positive correlation between the two miRNAs, consistent with co-transcription (*R* ^2^ = 0.77; *P* < 0.001, Fig. 1D).

### Decreased expression of miR-15a and miR-16-1 negatively correlates with *CCND1* mRNA expression in *Men1**^+/−^*** **mouse pituitary tumours**

**Figure 2:**
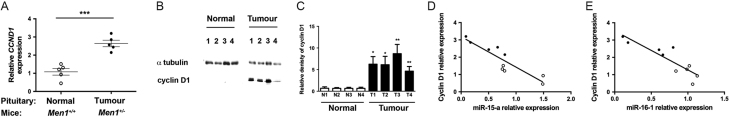
Correlation of miR-15a and miR-16-1 with *CCND1*. Analysis of five *Men1^+/^^−^* pituitary tumours (closed circles) and five WT pituitary samples (open circles) by qRT-PCR demonstrated a significant increase in expression of *CCND1*, a known target of miR-15a and miR-16-1, in the tumour samples (A, ***P < 0.0001). Cyclin D1 protein expression, encoded by *CCND1*, was evaluated in four tumours from *Men1^+/^^−^* mice and four normal pituitaries from WT mice (*Men1^+/+^*), using Western blot analyses. All four pituitary tumours expressed cyclin D1, whereas cyclin D1 was not detectable in the normal pituitary samples (B). Densitometry analysis of four Western blots confirmed that cyclin D1 expression is significantly higher in the four pituitary tumours (T1–T4 and filled bars) from *Men1^+/^^−^* mice compared to normal (N1–N4 and open bars) pituitaries from WT (*Men1^+/+^* mice) (C, *P < 0.05, **P < 0.005). All values were normalised to α-tubulin expression. A significant negative correlation was observed in the relative expression of both miR-15a (D) and miR-16-1 (E) compared to *CCND1* (D, *R* ^2^ = 0.81, *P* < 0.0005 and E, *R* ^2^ = 0.78, *P* < 0.001, respectively).

*CCND1* is reported to be regulated by miR-15a/miR-16-1 in prostate and non-small-cell lung cancer (Cimmino *et al.* 2005, Bonci *et al.* 2008, Calin *et al.* 2008, Bandi *et al.* 2009, Croce 2009, Salerno *et al.* 2009), and we therefore examined *CCND1* expression in the pituitary of the *Men1^+/^^−^* mice that had reduced expression of miR-15a and miR-16-1 (Fig. 1A, B and C). This revealed a significant increase in *CCND1* mRNA levels in *Men1^+/^^−^* pituitary tumours (*n* = 5), when compared to normal pituitaries (*n* = 5) from WT mice (2.6-fold, *P* < 0.0005, Fig. 2A). These findings were confirmed by Western blot (Fig. 2B) and densitometry analyses (Fig. 2C), which revealed that expression of the cyclin D1 protein was significantly higher (by 4.6-fold to 8.7-fold, *P* < 0.05–0.005) in the pituitary tumours than in those of the normal pituitaries. Moreover, there was a significant inverse correlation between the levels of *CCND1* mRNA and both miR-15a (Fig. 2D, *R* ^2^ = 0.81; *P* < 0.0005), and miR-16-1 (Fig. 2E, *R* ^2^ = 0.78, *P* < 0.001), thereby suggesting that *CCND1* mRNA may be under the direct regulation of miR-15a and miR-16-1. Analysis of the expression of a putative let-7a mRNA target, *KRAS* revealed significantly increased expression of *KRAS* (by 1.5 fold, *P* < 0.005, Supplementary Fig. 3A), in the pituitary tumours of *Men1^+/^^−^* mice, when compared to normal pituitaries of *Men1^+/+^* mice, although a significant inverse correlation was not observed between the expression of *KRAS* and let-7a (Supplementary Fig. 3B).

### Cyclin D1 expression is directly regulated by miR-15a and miR-16-1

**Figure 3:**
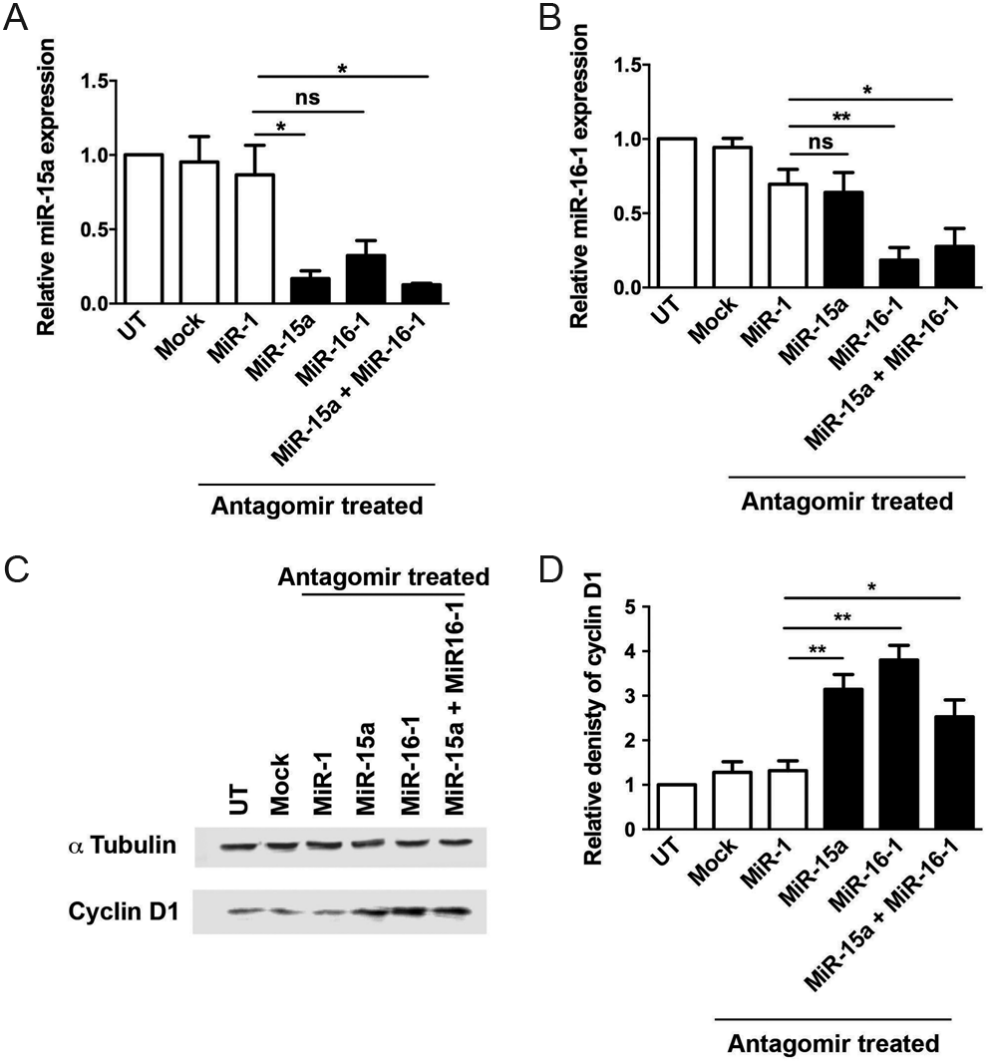
miR-15a and miR-16-1 antagomir treatment increases cyclin D1 expression in human HeLa cells. Inhibition of miR-15a and miR-16-1 binding to their target mRNAs was performed using antagomir transfections in HeLa cells. miRNA-15a levels were significantly reduced when HeLa cells were transfected with antagomirs targeting either miR-15a alone or against both miR-15a and miR-16-1 (*P < 0.05) (A). A significant decrease in miR-16-1 levels was also seen when cells were transfected with antagomirs targeting miR-16-1 alone or both miR-15a and miR-16-1 (*P < 0.05 and ** P < 0.01) (B). Forty-eight hours following antagomir transfection the levels of cyclin D1 were assessed using Western blot analyses, and α-tubulin was used as a loading control (C). Densitometry analysis of the Western blots (*n* = 4) revealed that cyclin D1 expression was significantly increased following transfection with antagomirs targeting either miR-15a, miR-16-1 alone or in combination (*P < 0.05, **P < 0.005) (D). UT, untransfected.

To further analyse the relationship between these miRNAs and cyclin D1, we altered the levels of miR-15a and miR-16-1 *in vitro* by transfecting HeLa cells with antagomirs that inhibited miR-15a and miR-16-1. Treatment with the miR-15a antagomir significantly decreased expression of miR-15a (14-fold (*P* < 0.05)), but not miR-16-1 (Fig. 3A), and treatment with the miR-16-1 antagomir significantly decreased miR-16-1 expression (12-fold (*P* < 0.005), but not miR-15a (Fig. 3B). miR-15a and miR-16-1 antagomir treatment also led to significant increases in cyclin D1 expression (3.1-fold (*P* < 0.05) and 3.8-fold (*P* < 0.005), respectively, Fig. 3C and D). Simultaneous transfection of HeLa cells with antagomirs to both miR-15a and miR-16-1 resulted in similar decreases in expression to that observed with single antagomir transfection (both *P* < 0.05, Fig. 3A and B), although the increase in cyclin D1 expression was lower in the co-transfected cells (2.4-fold, *P* < 0.05, Fig. 3C and D).

### Menin regulates the expression of miR-15a

**Figure 4:**
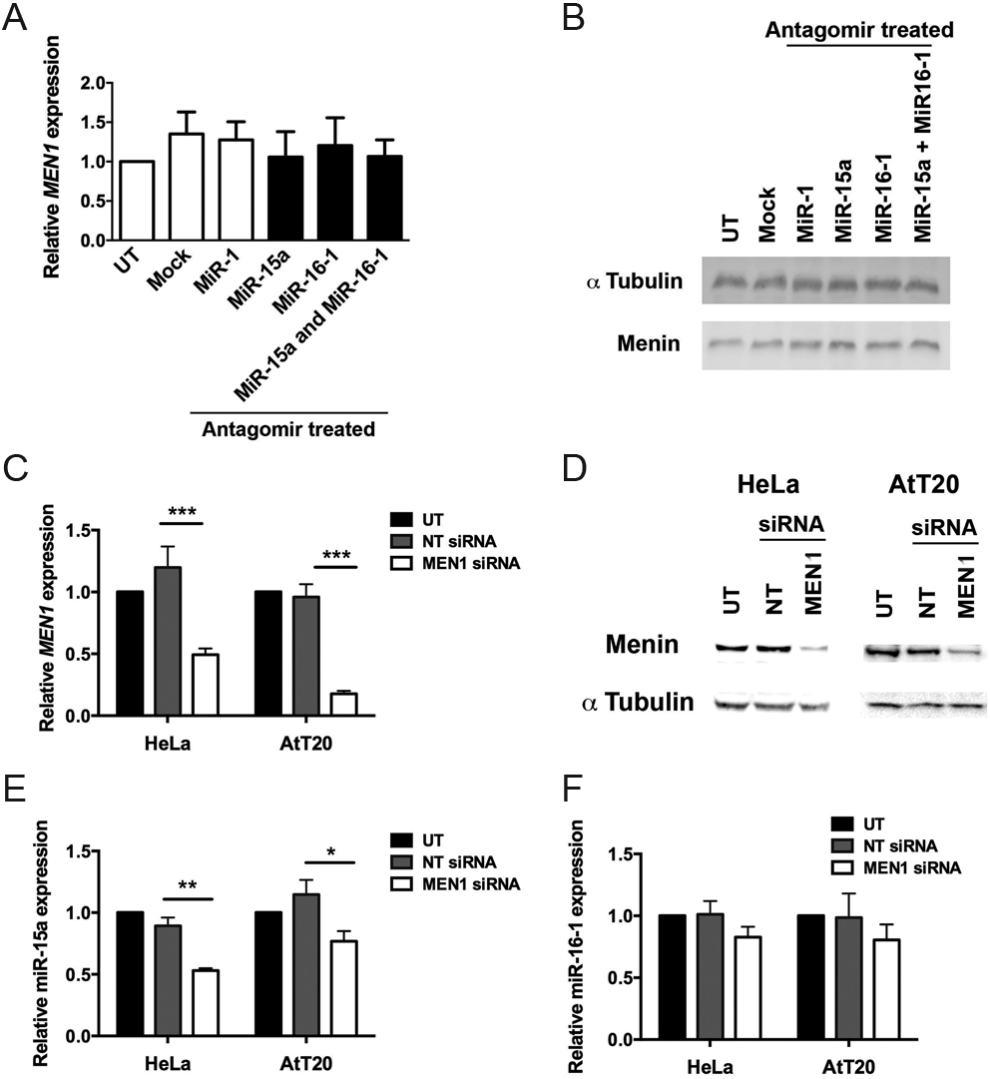
Relationship between miR-15a/miR-16-1 and menin in HeLa and AtT20 cells. Significant differences were not observed in the levels of *Men1* or menin expression after antagomir treatment of HeLa cells, evaluated by qRT-PCR (A) and Western blot (B), respectively. Use of *MEN1*-specific siRNA in HeLa and AtT20 cells decreased expression of *MEN1* (C) (assessed by qRT-PCR, ***P < 0.0005) and menin (D) (assessed by Western blot analysis). The decreased expression of *MEN1* and menin, caused by the specific *MEN1* siRNA, resulted in a significant decrease in the expression of miR-15a in both HeLa and AtT20 cells (E, **P < 0.005; *P < 0.05), but not in miR-16-1 expression (F). NT, non-targeting siRNA; UT, untransfected.

Menin has been reported to regulate, and to be regulated by miRNAs, including via feedback loops (Luzi *et al.* 2012*a*,*b*, Wang *et al.* 2013, 2014, Caplin *et al.* 2014, Gurung *et al.* 2014, Li *et al.* 2014, Vijayaraghavan *et al.* 2014, Lu *et al.* 2015, Ouyang *et al.* 2015, Ehrlich *et al.* 2017, Hou *et al.* 2017). To determine if there was a feedback loop present between menin and miR-15a/miR-16-1, we examined the effects of antagomir-induced inhibition of miR-15a or miR-16-1 on menin expression in HeLa cells using qRT-PCR and Western blot analyses. Antagomirs to miR-15a and miR-16-1 did not alter expression of the *MEN1* gene or menin (Fig. 4A and B), indicating that menin expression is not directly regulated by miR-15a or miR-16-1. To assess the possible role of menin in regulating miR-15a or miR-16-1 expression, menin knockdown experiments were performed in human HeLa cells, and in the mouse pituitary cell line AtT20, and miR-15a and miR-16-1 levels analysed using qRT-PCR. Knockdown of menin in HeLa and AtT20 cells, which was confirmed at both the mRNA (both *P* < 0.0005, Fig. 4C) and protein levels (Fig. 4D), resulted in a significant decrease in miR-15a expression (*P* < 0.005 and *P* < 0.05 respectively, Fig. 4E) but not of miR-16-1 (Fig. 4F). miR-15a and miR-16-1 expression are reported to be under the control of a promoter of their host gene, *DLEU2* (Lerner *et al.* 2009), but our analysis of *DLEU2* expression following menin knockdown in HeLa and AtT20 cells revealed that there were no alterations in *DLEU2* expression (Supplementary Fig. 4). This finding, which is consistent with the observation that the promoter region of *DLEU2* does not contain a menin-binding site (Scacheri *et al.* 2006), indicates that menin does not appear to regulate the expression of miR-15a by direct binding to the *DLEU2* promoter, but instead may influence expression via alternate mechanisms.

## Discussion

Our study has revealed that (1) the expression of the microRNAs miR-15a, miR-16-1 and let-7a are downregulated in pituitary tumours that develop in a *Men1^+/^^−^* mouse model; (2) there is a significant positive correlation between miR-15a and miR-16-1 expression; (3) the decreased miR-15a and miR-16-1 expression is associated with increased cyclin D1 expression and (4) that loss of menin expression is associated with a decrease in miR-15a expression.

The decreased expression of miR-15a, miR-16-1 and let7a in the pituitary tumours of *Men1^+/^^−^* mice is in agreement with the reported down regulation of these miRNAs in human pituitary tumours (Bottoni *et al.* 2005, 2007, Amaral *et al.* 2009). In addition, the positive correlation between miR-15a and miR-16-1 in the *Men1^+/^^−^* mouse pituitary tumours (Fig. 1) indicates that these miRNAs are likely transcribed as a polycistronic cluster, under the control of the same promoter elements, as reported for these miRNAs in patients with chronic lymphocytic leukaemia (CLL) (Calin *et al.* 2008). Moreover, in humans, the *miR-15a-miR-16-1* cluster is thought to act as a tumour suppressor and its chromosomal location (13q14) is a site of frequent allelic disruption or loss in several tumours including CLL, prostate cancer and pituitary tumours where its loss is associated with aggressive features (Pei *et al.* 1995, Hyytinen *et al.* 1999, Dong *et al.* 2001, Calin *et al.* 2002, 2008). These observations support a role for miR-15a and miR-16-1 in the aetiology of MEN1-associated pituitary tumours.

The observation of increased cyclin D1 protein expression in association with down regulation of miR-15a and miR-16-1 in pituitary tumours from *Men1^+/^^−^* mice (Fig. 2), suggests that cyclin D1 is a putative target of miR-15a and miR-16-1 in these *Men1-associated* pituitary tumours. In addition, transfection of HeLa cells with specific antagomirs to miR-15a and miR-16-1 significantly increased cyclin D1 expression (Fig. 3), thereby confirming that cyclin D1 is a likely target of these miRNAs, and this is in agreement with previous human and mouse studies which have reported that cyclin D1 overexpression occurs in a significant proportion of human sporadic pituitary tumours (Gazioglu *et al.* 2007) and that miR-15a and miR-16-1 can directly regulate cyclin D1 expression in osteosarcoma and CLL (Salerno *et al.* 2009, Cai *et al.* 2012), and that the miR-15a and miR-16-1-binding sites in the 3′ UTR of cyclin D1 are highly conserved across species (Deshpande *et al.* 2009). This negative correlation between the decreased expression of both miR-15a and miR-16-1 and the increased expression of *Ccnd1* in *Men1*-associated pituitary tumours (Fig. 2) was not observed to occur between *Kras* and let-7a (Supplementary Fig. 3). Previous studies of laryngeal and lung cancers have reported that let-7a can regulate the expression of *KRAS*, and that menin is involved in let-7a miRNA processing (Johnson *et al.* 2005, Long *et al.* 2009, He *et al.* 2010, Oh *et al.* 2010, Guan *et al.* 2011, Wang *et al.* 2013, Gurung *et al.* 2014). However, our findings suggest that let-7a does not regulate *Kras* in pituitary tumours of *Men1^+/^^−^* mice and that it may act via alternative targets.

Menin was found to cause a decrease in miR-15a levels in the pituitary tumours of the *Men1^+/^^−^* mice, and this is similar to reports showing that menin can negatively regulate the expression of miR-26a and miR-29b (Luzi *et al.* 2012*a*, Ouyang *et al.* 2015). In addition, menin has been reported to form a negative feedback loop with miR-24-1, which can mimic the second ‘hit’ of *MEN1* i.e. loss of the second* MEN1* allele (Luzi *et al.* 2012*b*) and that miRNAs miR-421, miR-24, miR-802, miR-17 and miR-762 can all regulate menin expression (Caplin *et al.* 2014, Vijayaraghavan *et al.* 2014, Wang *et al.* 2014, Lu *et al.* 2015, Ehrlich *et al.* 2017, Hou *et al.* 2017). However, our results, which revealed that antagomirs of miR-15a or miR-16-1 did not affect menin expression (Fig. 4), do not support the existence of a feedback loop between these miRNAs and menin.

We demonstrate that *in vitro* knockdown of menin in HeLa and AtT20 cells significantly reduced the expression of miR-15a, but not miR-16-1 (Fig. 4). In addition, expression of *DLEU2* which is regulated under the same promoter as the miR-15a-miR-16-1 cluster (Lerner *et al.* 2009) was not altered after menin knockdown (Supplementary Fig. 4). It has previously been reported that in HEK293 cells menin can bind to the arsenite resistance protein (ARS2), which is involved in stabilising capped primary miRNA transcripts and delivering them to the primary miRNA processing complex (Gurung *et al.* 2014). Furthermore, loss of menin resulted in reduced levels of mature let-7a miRNA, but did not affect primary miRNA levels (Gurung *et al.* 2014). Therefore, we hypothesise that the loss of menin in the pituitary tumours and cell lines in our study disrupts the activity of ARS2, leading to dysregulation of miR-15a-miR-16-1 miRNA processing. However, in the tumours from menin null mice we observed a significant decrease in both miR-15a and miR-16-1, expression (Fig. 1); but, in the *in vitro* menin knockdown studies, we only observed a decrease in miR-15a (Fig. 4). There are two possible explanations for this: first in the *in vitro* studies, we did not observe complete menin loss as is seen in the *Men1^+/^^−^* mouse tumours, and therefore the residual menin expression may be attenuating the phenotype or second the menin-null tumours may accumulate additional mutations that can further alter miRNA expression, potentially by modifying the allelic imbalance of miR-15a-miR-16-1 expression, as reported in CLL (Veronese *et al.* 2015). These studies in CLL have reported that expression of miR-15a and miR-16-1 shows allelic imbalance, with transcription of this cluster being simultaneously regulated by RNA polymerase (RP) II and RPIII mechanisms (Veronese *et al.* 2015). Thus, miR-15a-miR-16-1 could be transcribed by RPII as a capped primary miRNA sequence or after splicing as an uncapped transcript by RPIII and as ARS2 is involved in stabilising capped primary miRNA transcripts, the menin-ARS2 interaction would only be important for RPII-mediated transcription (Lee *et al.* 2004, Veronese *et al.* 2015).

In conclusion, we demonstrate that miR-15a and miR-16-1 are both downregulated in pituitary tumours of *Men1^+/^^−^* mice and that this decrease in expression correlates with an increase in cyclin D1 expression. Moreover, in human cells, inhibition of miR-15a and miR-16-1 binding to mRNA using antagomirs significantly increases cyclin D1 expression, and this may be due to altered processing of miR-15a by menin, as our menin-knockout studies revealed a significant decrease in miR-15a.

## Supplementary Material

Supporting Figure 1

Supporting Figure 2

Supporting Figure 3

Supporting Figure 4

## Declaration of interest

The authors declare that there is no conflict of interest that could be perceived as prejudicing the impartiality of the research reported.

## Funding

This work was supported by: the UK Medical Research Council grants G9825289, G1000467 (K E L, P J N, M S, R D, R V T) and G0601423 (P J N); AMEND Research Fund Award (K E L); a Wellcome Trust Senior Investigator Award (R V T); Royal Australasian College of Physicians Vincent Fairfax Family Foundation Research Fellowship (C J Y); Australia Awards Endeavour Postgraduate Research Fellowship Award (C J Y); Novartis Pharmaceuticals Australia Educational Grant (C J Y); Ipsen Pharmaceuticals Australia Educational Grant (C J Y) and The Unicorn Foundation Educational Grant (C J Y).

## Author contribution statement

K E L, P J N and R V T designed research; K E L, P J N, C J Y, M S, R D, G V W and M R B performed experiments; K E L, P J N and R V T wrote the manuscript and K E L, P J N, C J Y, M S, R D, G V W, M R B and R V T edited the manuscript.
