# Supplementary figures and images for: miR-15a/miR-16-1 expression inversely correlates with cyclin D1 levels in Men1 pituitary NETs
Source: J Endocrinol. 2018 Sep 28;240(1):41–50. doi: 10.1530/JOE-18-0278 (PMC6347280; doi:10.1530/JOE-18-0278)

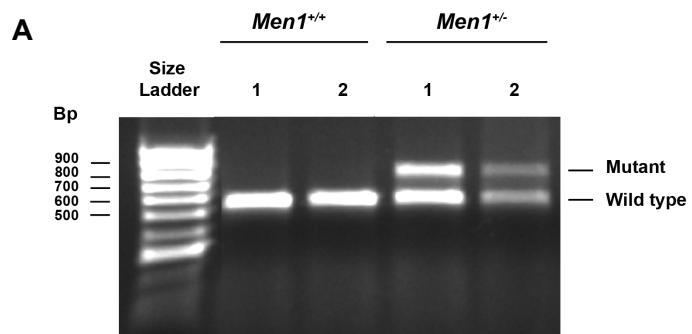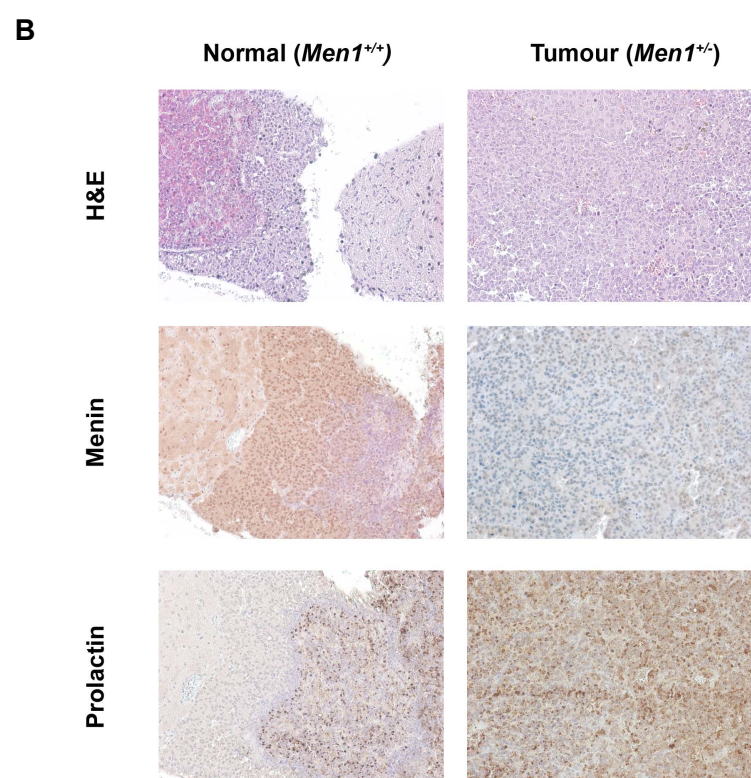

Supplement: Supporting Figure 1 [file JOE-18-0278-s001.pdf]

**A**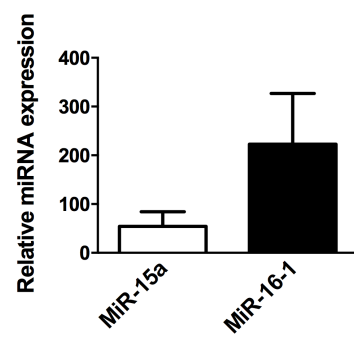**B**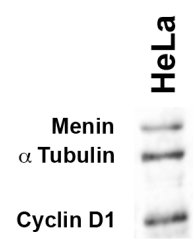

Supplement: Supporting Figure 2 [file JOE-18-0278-s002.pdf]

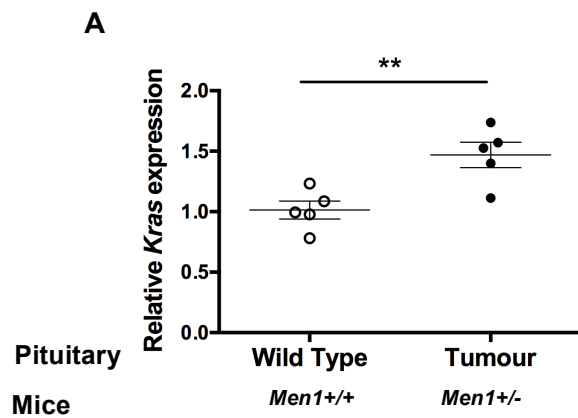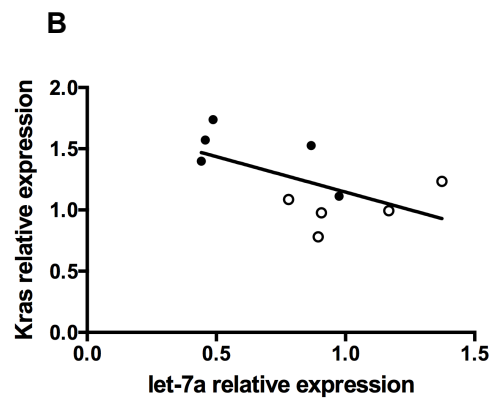

Supplement: Supporting Figure 3 [file JOE-18-0278-s003.pdf]

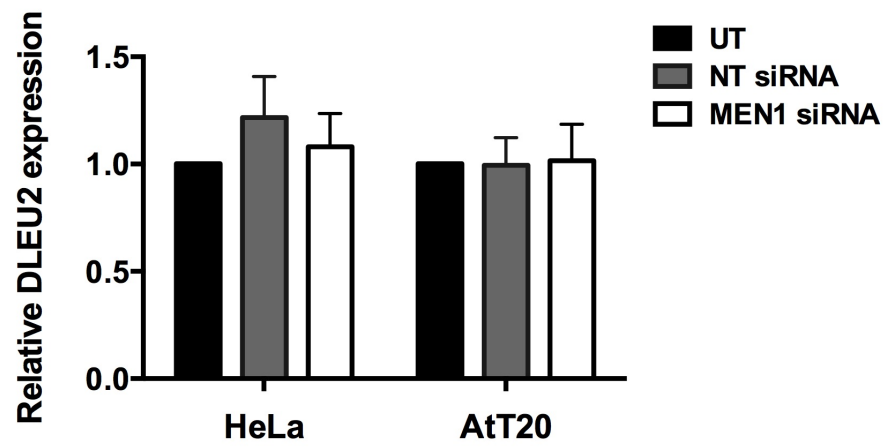

Supplement: Supporting Figure 4 [file JOE-18-0278-s004.pdf]
